# Therapeutic effects of topical Mycophenolate mofetil on hydroquinone-induced depigmentation in Guinea pigs and mice

**DOI:** 10.1080/07853890.2025.2544880

**Published:** 2025-08-11

**Authors:** Yuan Guo, Yi-fei Yang, Yanhua-Li, Yun Zhang, Haixia-Shi

**Affiliations:** a Department of Dermatology, The Affiliated Hospital of Yangzhou University, Yangzhou University, Yangzhou, China; b Department of Dermatology, The Affiliated Hospital of Jiangsu University, Zhenjiang, China; c Laboratory for Regeneration Medicine, Jiangsu University, Zhenjiang, China

**Keywords:** Melanogenesis, mycophenolate mofetil, WNT

## Abstract

**Background:**

Vitiligo is a depigmentation disorder with an undefined specific pathogenesis, with the autoimmune theory being a prominent etiology. Mycophenolate mofetil (MMF), a novel immunosuppressant, selectively inhibits inosine 5-monophosphate dehydrogenase (IMPDH), crucial for lymphocyte DNA synthesis. Recent research has highlighted MMF’s efficacy in vitiligo treatment; however, the precise mechanism remains unclear. This study aimed to explore the therapeutic impact and mechanism of vitiligo.

**Methods:**

We enrolled guinea pigs and C57/BL6 mice which were randomly divided into the hydroquinone group, the MMF & hydroquinone group, and the control group. Skin biopsy specimens were collected from designated areas which were stained using hematoxylin and eosin (H&E), Masson-Fontana (M-F), and immunofluorescence techniques. To elucidate the pharmacological mechanisms underlying the effects of MMF, real-time polymerase chain reaction (PCR) and western blotting analyses were performed.

**Results:**

A comparative analysis revealed a notable enhanced pigmentation in the MMF-conjunction group. Subsequent to MMF application, there was a significant augmentation in both the number of melanocytes within the basal layer and melanin-containing hair follicles. MMF activated tyrosinase (TYR), which had been suppressed by hydroquinone, and led to an upregulation of melanin-associated genes such as TYR, TYRP-1, MITF, and SILV. Additionally, there was an increase in WNT pathway-related proteins, including β-catenin, GSK3β, Axin2, Dkk, and Dact2. MMF effectively mitigated the vitiligo-like increase in IFN-γ induced by hydroquinone safely.

**Conclusion:**

MMF exhibits a notable capacity to ameliorate the pigment-loss effect associated with hydroquinone through the activation of the WNT signaling pathway. Consequently, MMF emerges as a promising therapeutic agent for mitigating skin pigmentation.

## 1. Introduction

WNT signaling plays a pivotal role in facilitating the differentiation of melanocyte precursors within the skin. Elevated levels of oxidative stress, observed in melanocytes of vitiligo patients, have the potential to directly impede the activation of the WNT pathway [1]. Furthermore, disruptions in this signaling cascade hinder melanocyte regeneration and repigmentation, thereby exacerbating the depigmentation associated with vitiligo [2]. Notably, the application of pharmacological agents capable of activating the WNT pathway in *ex vivo* patients of vitiligo skin has successfully induced the differentiation of resident stem cells into pre-melanocytes [3]. Consequently, the WNT pathway emerges as a critical factor in the pathogenesis and progression of vitiligo. Based on these insights, we hypothesize that therapeutic strategies aimed at activating the WNT pathway could represent a promising approach to enhance melanocyte regeneration.

Mycophenolate mofetil (MMF), a purine antagonist, exhibits selective inhibitory effects on the proliferation of activated lymphocytes and diminishes the migration of lymphocytes and monocytes towards inflammatory sites [4]. Additionally, MMF plays a pivotal role in modulating the WNT signaling pathway. Ryu and colleagues [5] suggested that MMF reverses IFN-γ-induced catagen-like alterations through the activation of the WNT/β-catenin signaling cascade.

MMF serves as an immunomodulatory agent due to its potent anti-inflammatory properties. Jeong et al. [6] investigated the impact of mycophenolic acid on dermal papilla cell proliferation and anagen hair follicle induction. Nevertheless, the comprehensive understanding of MMF’s beneficial effects in vitiligo remains incomplete. Nasrin Saki et al. conducted a study involving 30 patients with localized vitiligo, administering a topical treatment of 18% MMF twice daily for a duration of three months. Notably, at the conclusion of the study, 36.6% of the patients exhibited approximately 25% repigmentation of their lesions, with no reported adverse effects [7]. In sunmmary, the specific impact of MMF on melanin-associated disorders and the potential mediation of this effect through WNT activation are yet to be elucidated.

A unique advatage of MMF over other medicine lies in its niche as a relapse-preventive agent rather than a rapid repigmentation inducer, suggesting its optimal use in combination therapies [8].

Chemical decolorization by hydroquinone has been used to establish a stable animal model of vitiligo [9]. In this research endeavor, our objective was to investigate the impact of MMF on animal models exhibiting HQ-induced depigmentation and to delve into the underlying mechanisms through which MMF exerts its effects in vitiligo.

## 2. Materials and methods

### 2.1. Ethics approval

This study received approval from the Laboratory Animal Medical Ethics Committee of of Jiangsu University and adhered strictly to international ethical standards, as well as the guidelines outlined in the National Institutes of Health’s Guide for the Care and Use of Laboratory Animals (NIH publication No. 85Y50, revised 1996), ensuring the highest level of ethical conduct throughout the research process. The ethical approval number is: UJS-LAER-2017122101.

### 2.2. Chemicals reagents and machines

Mycophenolate mofetil was sourced from Nanjing Fcmacs Biotechnology Co., Ltd.

To formulate an 18% MMF cream, 9 grams of MMF were initially dissolved in 5 grams of alcohol and subsequently diluted in 45 grams of Eucerin cream, which was acquired from Shanghai Maclean Biochemical Technology Co., Ltd. Additionally, Masson-Fontana melanin stain was obtained from Beijing Suo Laibao Technology Co., Ltd., while Alexa Fluor^®^ 488-labeled goat anti-rabbit IgG (H + L) was procured from Nanjing Fcmacs Biotechnology Co., Ltd. The anti-tyrosinase rabbit polyclonal antibody was purchased from Abcam Biotechnology Co., Ltd. For imaging purposes, an immunofluorescence confocal scanning microscope (ECLIPSE Ti) was acquired from Nikon Corporation, Japan. Furthermore, a fluorescence quantitative PCR instrument (Stratagene Mx3000P) was sourced from Shanghai Jitai Biotech Co., Ltd. Western blotting was conducted utilizing appropriate chemiluminescence reagents. Serum levels of alanine aminotransferase (ALT, U/L), aspartate aminotransferase (AST, U/L), blood urea nitrogen (BUN, μmoI/L), Blood creatinine (B-CREA, μmoI/L), and Creatine Kinase-MB(CK-MB, μmoI/L) were measured using a fully automated biochemical analyzer (Roche Cobas^®^ 8000, Switzerland) with commercial kits (Roche Diagnostics, Basel, Switzerland; ALT: Cat# 04460785, AST: Cat# 04460773, BUN:Cat# 04460732, B-CREA: Cat# 04460716; CK -MB:Cat# 07092747).

### 2.3. Animals

Male guinea pigs, weighing 400 ± 50 grams, and 6-week-old male C57/BL6 mice were sourced from Yizheng Annex Technology Co. Ltd. The reason for selecting 6-weeks-old mice is that it conforms the hair growth cycle of C57/BL6 mice whose hair growth period lasted about 14-21 days, and rest period about 20–30 days (total cycle was about 5–6 weeks) [10]. These animals were housed in the Animal Laboratory of Jiangsu University under controlled conditions of 25 °C and 70% humidity, with ad libitum access to food and water. A total of fifty-four male guinea pigs were randomly assigned to three groups, each comprising 18 animals. The dorsal hair, covering an area of 6 × 9 cm, was symmetrically removed using a 1:1 mixture of rosin and paraffin, centered along the spine. At the same time, we used self-controlled study for guinea pigs. The right side was the treated side while the left side serving as the control side with no treatment for comparative observation. The shaved space of C57/BL6 mice was 3 × 3 cm which weren’t used self-controlled study. The experimental design involved dividing both the guinea pigs and mice into three distinct groups. The first group received hydroquinone monotherapy, the second group was administered a combination therapy of hydroquinone and MMF, and the third group served as the untreated control. Subsequently, the animals underwent the following treatments for a total duration of 50 days: (1) the untreated control group received four daily applications of physiological saline on the hair-depilated skin area; (2) the vitiligo model group was treated with twice-daily applications of 5% hydroquinone interspersed with two applications of normal saline [11]; and (3) the MMF-treated vitiligo group received a regimen of twice-daily applications of both 5% hydroquinone and 18% MMF on the depilated skin. Following the final administration, the mice were euthanized four hours later, and blood samples along with skin tissues were collected. All procedures involving animal handling adhered to the guidelines stipulated by the Animal Management Ordinance of the Chinese Ministry of Health, and the animal experimentation protocols were approved by the Animal Management Committee of the Laboratory Animal Center of Jiangsu University.

### 2.4. Visual observation of the percentage of the bleached area

Upon completion of the drug administration phase, the therapeutic efficacy of MMF in the experimental vitiligo animal model was assessed through visual inspection. The criteria for evaluation involved capturing images of the animals under consistent lighting conditions and from a standardized distance. Subsequently, these images were analyzed using Image-Pro Plus software to determine the percentage of depigmented area on the hair-removed skin.

### 2.5. Skin biopsy

On days 30, 40, and 50, skin biopsies were collected from designated areas using scissors. Immediately following procurement, the biopsy specimens were fixed in 10% formalin and subjected to routine processing procedures. They were then embedded in paraffin blocks, sectioned at a thickness of 8 micrometers using a standard microtome, and stained with H&E, M-F, and immunofluorescence for subsequent analysis.

### 2.6. Histopathology

#### 2.6.1. H&E staining

To verify the structural integrity of the tissues, routine H&E-stained sections were meticulously examined. The number of melanin-laden hair follicles within a single visual field was enumerated using a light microscope at a magnification of ×100. This counting process was independently repeated across ten distinct fields of view, and the average count was subsequently calculated to ensure accuracy and reliability.

#### 2.6.2. M-F staining

To assess the melanin density within melanocytes and keratinocytes, M-F staining was conducted. In brief, formalin-fixed tissue sections were exposed to ammoniacal silver nitrate solution in a sealed container for 15 min at a temperature of 60 °C. Following this, the samples were thoroughly rinsed in distilled water and subsequently treated with a reducing agent (hypo-solution) for 5 min. Counterstaining was achieved using neutral red for an additional 5 min, followed by rinsing with distilled water. The preparations were then subjected to dehydration using absolute alcohol. At a high magnification of ×400, the number of basal melanocytes within a single visual field was counted, and this process was repeated across ten distinct fields of view to determine the average cell count.

#### 2.6.3. Immunofluorescence staining

For immunostaining of tissue sections, cells were fixed with 4% paraformaldehyde sourced from Solarbio (Beijing, China) for 15 min. Subsequently, immunofluorescence staining was performed using specific primary antibodies directed against TYR (diluted 1:100; EMD Millipore, Billerica, MA) and TYRP-1 (diluted 1:200; EMD Millipore, Billerica, MA). Alexa Fluor 488-conjugated goat anti-mouse IgG2a secondary antibody (diluted 1:500; Life Technologies, Carlsbad, CA) was employed for detection, and the nuclei were counterstained with DAPI.

The immunostaining for the melanin-function gene product TYR served as a marker to confirm the presence of melanocytes and to verify the concordance of staining patterns with the aforementioned stains.

### 2.7. Biochemical measurements

Routine blood analysis was conducted to assess alterations in white blood cell and lymphocyte counts, aiming to validate the protective role of MMF in modulating the WNT pathway through the reduction of T lymphocytes. Additionally, liver and kidney function tests were performed to ensure the safety profile of MMF administration.

Serum was separated by centrifugation at 3,000 × g for 15 min at 4 °C. All assays were performed at 37 °C with absorbance measured at 340 nm for ALT, AST, BUN, B-CREA, CK-MB. Each batch included triplicate measurements and internal quality control samples to ensure inter-assay CV <5%.

### 2.8. Real-time PCR and Western-blot

Skin tissue samples measuring 2 × 2 cm were collected from the center of the drug delivery site. Total RNA was extracted using TRIzol reagent (Thermo Fisher Scientific, Carlsbad, CA) and subsequently reverse transcribed into cDNA with a RevertAid First Strand cDNA Synthesis Kit (Thermo Fisher Scientific, Carlsbad, CA), adhering to the manufacturer’s protocols. Real-time qPCR was executed on the Stratagene Mx3000P platform using SYBR Premix Ex Taq (Takara, Beijing, China). Gene expression levels were normalized against GAPDH, serving as the internal reference gene. The primer sequences utilized are detailed in Supplementary Table 1. Western blot analysis was conducted on skin tissue lysates containing 20 μg of protein per sample, employing specific antibodies targeting GAPDH, β-catenin, phospho-β-catenin, GSK3β, and phospho-GSK3β (Sigma-Aldrich, St. Louis, MO, USA). Protein expression was detected by chemiluminescence.

### 2.9. Data analysis

The results are presented as the mean ± SD, based on the number of guinea pigs and mice in each experimental group (*n*). The overall statistical significance of the findings was evaluated using one-way ANOVA through the SPSS software version 16.0. A *p*-value of less than 0.05 was considered statistically significant.

## 3. Results

### 3.1. Establishment of a depigmented Guinea pig model using hydroquinone

**Figure 1.**
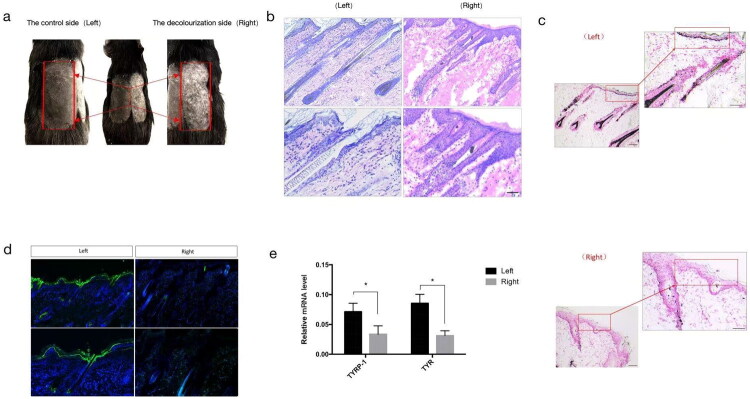
Vitiligo decolourization model and its identification. (a) Morphology of hydroquinone-induced pigmentation-loss spots on the right side of Guinea pig hair-removed skin. (b) H&E staining results of the left and right lesions. (c) Masson-fontana staining shows the number of the melanin-containing hair follicles and melanocytes on the basal layer on the left and right portions. (d) Immunostaining for the tyrosinase marker. (e) Quantitative PCR results show the expression of melanin-related gene markers in the left and right lesions. (sample size is 18 for (a)–(e)) (*p < 0.05).

Thirteen days post-application of a 5% hydroquinone topical solution, a limited number of discrete depigmented spots emerged exclusively on the right-side exposed skin, contrasting with the unaffected left side. Subsequently, by the 30th day, these depigmented areas progressively coalesced into plaques (Figure 1a). Histological assessments, incorporating hematoxylin and eosin (H&E) staining and Masson-Fontana staining, revealed an absence of melanin-laden hair and melanocytes within the basal layer on the right side, whereas these structures remained intact on the left (Figure 1b and c). Immunofluorescence staining was employed to detect the specific melanocyte marker protein, TYR (Figure 1d). Furthermore, the expression levels of genes characteristic of mature melanocytes, namely TYR and TYRP-1, were notably downregulated in the affected right-side lesions compared to the unaffected left side (*p < 0.05) (Figure 1e).

### 3.2. The role of MMF in the vitiligo decolonization model

**Figure 2.**
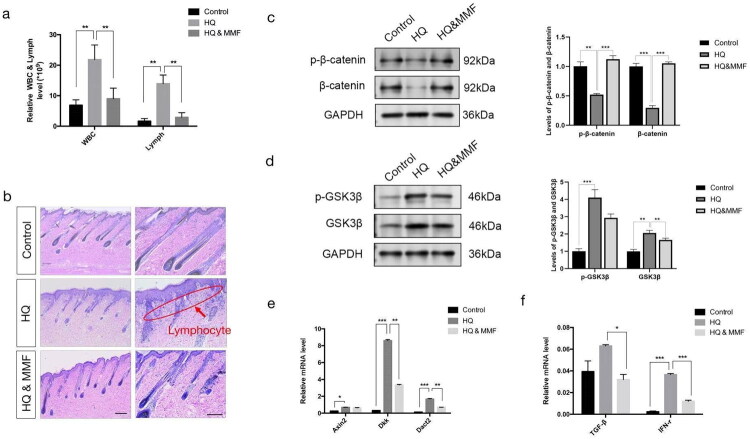
The role of MMF in hydroquinone-induced Guinea pig models. (a) Morphology of the decolorization areas on the 30th 40th and 50th day in the decolorization group and the decolorization and MMF groups. (b) Observation of decolorized areas on the 30th, 40th and 50th days under wood’s light. (c) and (d) Image-pro plus calculation results for sunlight and under wood’s light. (e) H&E staining and Masson-Fontana staining of the control, decolorization, and decolorization and MMF groups. (f) Number of melanin-containing hair follicles under a 40x microscope. (*n* = 10). (g) Number of melanocytes in the basal layer under the 400x microscope. (*n* = 10). (h) Quantitative PCR results showing the expression of melanin-related gene markers in the three groups. (i) Immunostaining for tyrosinase markers. (sample size is 18 for (a)–(i)) (*p < 0.05, ***p < 0.001).

When compared to the hydroquinone group, a marked reduction in the decolorization area was observed in the MMF group on the 30th, 40th, and 50th days (Figure 2a). Changes in the depigmented areas were visualized using Wood’s light (Figure 2b). Additionally, the Image-Pro Plus software was utilized to quantify the percentage of the depigmented area relative to the hair-removed portion, confirming the trends observed (*p < 0.001) (Supplementary Figures 1a and 2a, Figure 2c and d). To differentiate the pigmentation patterns among the three groups, skin tissues were collected on the 50th day for histological examination. In contrast to the control group, the hydroquinone group exhibited an absence of melanin-containing hairs and melanocytes in the basal layer as demonstrated by H&E and Masson-Fontana staining. Notably, MMF treatment led to melanin regeneration, evident from an increased presence of melanin-containing hairs and melanocytes in the basal layer compared to the decolorization group (Figure 2e). Statistical analysis revealed significant differences in the number of melanin-containing hairs and melanocytes among the three groups (*p < 0.05; ***p < 0.001) (Figure 2f and g). Furthermore, the expression levels of genes indicative of mature melanocytes, such as TYR and TYRP-1, were comparable between the hydroquinone and MMF groups, and both were higher than those in the hydroquinone-only group when compared to the control group (*p < 0.05) (Figure 2h). Immunofluorescence staining also confirmed the presence of the specific melanocyte marker protein, TYR (Figure 2i). Collectively, these findings underscore the protective effect of MMF against hydroquinone-induced depigmentation.

### 3.3. Detection of cytokine changes in the WNT pathway by RT-PCR and Western blotting

**Figure 3.**
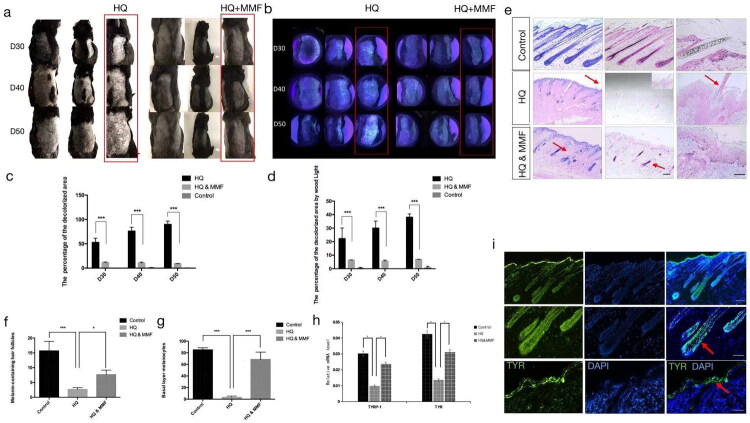
Expression of WNT pathway-related cytokines by RT-PCR & Western blotting. (a) Expression levels of white blood cells and lymphocyte cells in the serum of the control group, the hydroquinone group and the hydroquinone & MMF group. (b) H&E staining shows the distribution and the number of the lymphocytes in the three groups. (c), (d) Analysis of WNT signaling-related protein expression (β-catenin, GSK3β) in the three groups. (e) Quantitative PCR results show the expression of the WNT pathway genes (Axin2, dkk, Dact2) in the different groups. (f) Quantitative PCR results show the expression of the WNT-related genes (TGF-β, IFN-γ) in the different groups. (sample size is 18 for (a)–(f)) (*p < 0.05, **p < 0.01, ***p < 0.001).

Subsequently, we conducted a comprehensive blood analysis to assess the immune status of the three groups. The results revealed a substantial elevation in white blood cells and lymphocytes following hydroquinone exposure, which was mitigated by the administration of MMF (*p < 0.01) (Figure 3a). Consistent with these findings, H&E staining demonstrated a similar lymphocyte distribution across all groups (Figure 3b). To delve into the underlying mechanisms of immune variations among the groups, we employed western blotting to quantify the protein levels of β-catenin and GSK-3β within the WNT signaling pathway (*p < 0.05; **p < 0.01; ***p < 0.001) (Figure 3c and d). Additionally, real-time PCR was conducted to examine the expression trends of genes involved in the WNT pathway, including Axin2, DKK, and Dact2, as well as WNT-associated genes such as TGF-β and IFN-γ (*p < 0.05; **p < 0.01; ***p < 0.001) (Figure 3e and f). Overall, compared to the control group, hydroquinone treatment significantly upregulated the molecular components of the WNT pathway, while co-treatment with MMF attenuated this effect.

### 3.4. Confirming the safety of MMF through biochemical indicators

**Figure 4.**
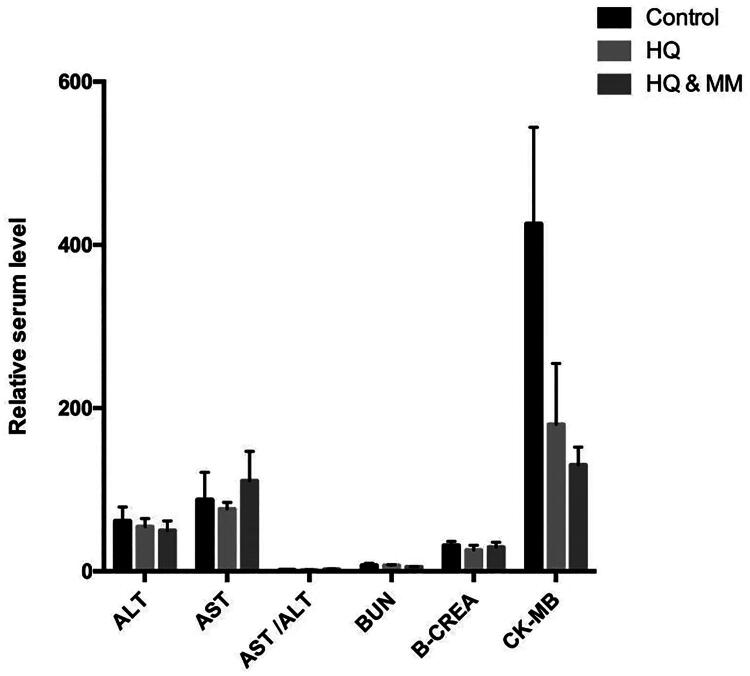
The safety of MMF assessed through biochemical indicators. The liver and kidney function of Guinea pigs. (sample size is 18) (*p* > 0.05).

We conducted an assessment of liver and kidney function in the peripheral blood of guinea pigs and found no statistically significant differences in the liver and kidney function indices among the MMF, hydroquinone, and control groups (*p > 0.05), (Figure 4a).

### 3.5. Effect of MMF on the hair vitiligo model

**Figure 5.**
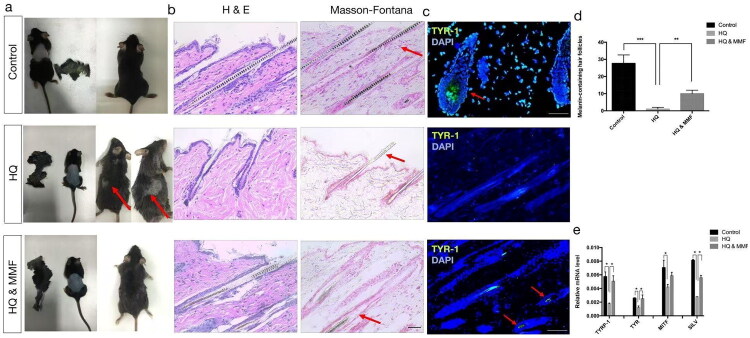
Effect of MMF on the hair vitiligo model. (a) Morphology of the white hair areas on the 30th day in the control group, the decolourization group and the decolorization & MMF group. (b) H&E staining and Masson-Fontana staining of the different groups. (c) Immunostaining for tyrosinase markers in the three groups. (d) Number of the melanin-containing hair follicles under a 40x microscope. (*n* = 10). (e) Quantitative PCR results show the expression of melanin-related genes in the three groups. (sample size is 18 for (a)–(e)) (*p < 0.05, **p < 0.01, ***p < 0.001).

In our hair vitiligo model, we employed an identical approach to validate the role of MMF in a HQ-induced pigment-loss model. The outcomes are summarized as follows: Upon topical application of HQ, white hair patches emerged on the 14th day in the depigmentation group, whereas no such patches were observed in the MMF-treated group (Figure 5a). Histological examination *via* H&E staining showed a notable reduction in melanin-containing follicles in the depigmentation group, whereas an increase was observed in the MMF group (Figure 5b). Masson-Fontana staining, which highlights melanin distribution, further demonstrated localized pigmentation in the MMF group, contrasting with the lack of pigmentation in the HQ-treated group (Figure 5b). To confirm the reappearance of melanin with MMF treatment, an immunofluorescence staining experiment was conducted, yielding positive staining with specific antibodies recognizing the human nucleus and the melanocytic marker TYRP1 (Figure 5c). Statistical analysis revealed significant differences in melanin-containing hair among the three groups (**p < 0.01; ***p < 0.001) (Figure 5d). Additionally, real-time PCR was performed to analyze melanocyte-related genes, including TYR, TYRP-1, MITF, and SILV, confirming consistent results (*p* < 0.05) (Figure 5e). Collectively, these findings underscore the capacity of MMF to counteract the inhibitory effects of HQ on tyrosinase and melanogenesis-related signaling pathways.

## 4. Discussion

Normalizing melanocyte stress, inhibiting autoimmunity, and fostering melanocyte regeneration constitute the cornerstone of an effective vitiligo treatment strategy. Various immunosuppressive agents have been demonstrated to enhance melanocyte deposition and differentiation. Inhibitors of the phosphatase calcineurin, such as pimecrolimus and tacrolimus, have been found to suppress lymphokine production and interleukin release, thereby reducing effector T-cell function [12]. Pimecrolimus demonstrates comparable efficacy and a superior safety profile compared to topical corticosteroids [13]. Fontas et al. [14] reported that twice-weekly application of 0.1% tacrolimus ointment effectively prevented depigmentation of vitiligo patches and promoted repigmentation. Furthermore, rapamycin offers promising approaches for vitiligo management by modulating mTORC1 and NF-κB pathways to induce regulatory T cells (Tregs) [15]. Bishnoi [16]. highlighted mycophenolate mofetil as a crucial stabilizing agent capable of halting actively progressing vitiligo. However, the precise mechanisms underlying melanocyte growth stimulation by immunosuppressant medications remain elusive.

In this study, we established a hydroquinone-induced pigment-loss model to explore the therapeutic potential of topical mycophenolate mofetil (MMF) in melanin-loss diseases. Our findings revealed that MMF counteracts the depigmenting effects of hydroquinone through direct interaction with tyrosinase, providing novel evidence that immunosuppressant medications play a pivotal regulatory role in melanin growth [17]. We further delved into the therapeutic mechanisms of MMF.

Currently, a stable vitiligo animal model has been developed through chemical depigmentation. Hydroquinone inhibits tyrosinase and melanin production-related signaling pathways, disrupting the catalysis and conversion of tyrosine, a rate-limiting enzyme in melanin synthesis, by TYRP-1. To investigate the *in vivo* functions of MMF, we employed a pigment-loss model induced by hydroquinone to assess melanin reconstitution [18]. Hydroquinone, as a tyrosinase and melanogenesis-related signaling pathway inhibitor, impedes the TYR-catalyzed conversion of tyrosine, a crucial step in melanin synthesis [11].

Morphological assessments under sunlight and Wood’s light showed that depigmentation spots coalesced into plaques after hydroquinone treatment but disappeared with MMF application. Image-Pro Plus software was used to quantify depigmentation areas [19]. H&E and Masson-Fontana staining revealed that MMF-induced melanocytes and their melanin products were predominantly localized in the basal layer and hair bulb, which are typical sites for mature melanocytes in both guinea pigs and mice [20–22]. Additionally, MMF upregulated the expression of melanin-specific genes TYR and TYRP-1, which are essential for tyrosinase activity [23,24]. In C57/BL6 mice, the MMF group exhibited increased levels of microphthalmia-associated transcription factor (MITF) and Premelanosome Protein (SILV), known regulators of melanocyte differentiation, pigmentation, proliferation, and survival [25,26]. Histological analysis showed an increase in melanin-containing hair follicles and basal melanocytes after MMF treatment [27]. Perifollicular repigmentation, primarily dependent on hair follicle melanocytes, is a common feature of vitiligo treatment response [28]. MMF application resulted in melanocytes containing melanin particles in the hair follicles of C57/BL6 mice. In guinea pigs, melanocytes are found in basal cells, hair follicles, and keratinocytes of the middle layer [29].

We hypothesize that MMF blocks HQ-induced IFN-γ upregulation by activating the WNT/β-catenin signaling pathway, thereby enhancing the expression of β-catenin target genes such as Axin2, DKKs, and Dact-2. This aligns with the recognized importance of Wnt/β-catenin signaling in melanocyte growth and differentiation. Furthermore, MMF suppressed TGF-β, a potent melanin-loss-promoting factor in mice [30].

WNT signaling, crucial for the differentiation of melanocyte precursors in the skin, is defective in vitiligo patient melanocytes. Impaired WNT signaling contributes to disease pathogenesis and inhibits melanocyte regeneration during treatment. Conversely, Regazzetti et al. [31] proposed that WNT activators may promote melanocyte differentiation and repigmentation. Thus, therapeutic WNT activation emerges as a promising melanocyte regeneration therapy for vitiligo [1]. Our results indicate that MMF restores melanin characteristics in the follicular and basal layers and prevents HQ-induced melanin loss by activating the Wnt/β-catenin signaling pathway. This supports the growing recognition of Wnt/β-catenin signaling’s pivotal role in murine hair and melanocyte development. Our findings suggest that IFN-γ activates GSK3β to inhibit β-catenin activity in follicular cells and skin, inducing vitiligo-like pigment loss in models.

GSK3β is a crucial protein in Wnt/β-catenin signaling modulation. In the absence of WNT activation, β-catenin undergoes GSK-3β-mediated phosphorylation in the cytoplasm, leading to ubiquitination and proteasome destruction. Conversely, Wnt proteins interacting with Frizzled family receptors negatively regulate GSK3β, allowing β-catenin nuclear translocation and binding to MITF and LEF1 promoters, resulting in MITF and TYR transcriptional activation [2]. Recent research shows that the GSK3β inhibitor BIO enhances alkaline phosphatase (ALP) and insulin-like growth factor 1 (IGF-1) expression, indicators of hair follicle induction in dermal papilla cells (DPCs) [32]. Our study revealed that MMF stabilizes β-catenin by inhibiting GSK3β, upregulating β-catenin target genes such as Axin2, DKKs, and Dact-2, potentially mitigating melanin loss induced by IFN-γ [33–35]. Finally, we confirmed that MMF has no adverse effects on liver and kidney function in animal models. Further investigations are essential to elucidate the mechanistic role of IFN-γ in vitiligo pathogenesis, but MMF’s potential to counteract IFN-γ-mediated melanin loss suggests it as a highly promising vitiligo therapeutic agent.

This study has several limitations. While the murine models offer advantages in preclinical research, its translational value may be constrained by heterogeneity. Parallel studies using humanized models or clinical specimens are warranted to confirm these observations about the treatment of MMF in vitiligo.

## 5. Conclusions

In conclusion, our study demonstrated that MMF holds potential for melanocyte reconstitution, suggesting its role as a safe and efficacious topical therapy for depigmentation in patients. These findings offer valuable insights into the pathogenesis of vitiligo. Consequently, further exploration of MMF is necessary to ascertain its capacity to slow, arrest, or reverse melanin depletion in vitiligo. Additionally, elucidating the mechanism by which MMF activates melanocytes and facilitates their migration to the periphery warrants investigation.

## ARRIVE guidelines

The authors have adhered to ARRIVE guidelines.

## Supplementary Material

Supplemental Material

Author Checklist Full.pdf

## Data Availability

The datasets generated or analysed during the study are available from the corresponding author on reasonable request.
