# Supplementary material for: Therapeutic effects of topical Mycophenolate mofetil on hydroquinone-induced depigmentation in Guinea pigs and mice
Source: Ann Med. 2025 Aug 11;57(1):2544880. doi: 10.1080/07853890.2025.2544880 (PMC12344676; doi:10.1080/07853890.2025.2544880)
Supplement: Supplemental Material [file IANN_A_2544880_SM9710.docx]

**Supplementary Table 1. List of primers for real-time RT-PCR**

| **Gene** | **Forward** | | **Reverse** |
| --- | --- | --- | --- |
| **Tyr** | **TGTCTCAAGTACAAGGATCAGCCAAC** | | **GGTGTCTTCGGAGCCACTGTTC** |
| **Tyrp-1** | **TGGTGTAGTGACGGCAGACTCTAG** | | **GCATTGGCACGTCCTATTGAAGAATC** |
| **Gapdh** | **GAAGGTGGTGAAGCAGGCATCAG** | | **TGCTGTAGCCGAACTCATTGTCATAC** |
| **Axin2** | **TGCCACCAGCAGTACCACTCC** | | **TCCGCCTCAATCTCCTCCTTGG** |
| **Dkk** | **GCGGAGGACGAGGAGTGCAG** | | **CGGCAGGCGAGGCAGACC** |
| **Dact2** | **CCGTCAGGAGGCCAGGAGTG** | | **CCGAAGCAGTTGGTGGTGTGG** |
| TGF-β | **TCTCGGCTGCGGCTCCTG** | | **GATGGCCTCGATGCGCTTCC** |
| IFN-γ | **CAACAGCAGCAACAAGGTGCAG** | | **TCCTCTGGTTCGGTGACAGGTC** |
| **tyr** | **GGGCCCAAATTGTACAGAGA** | | **ATGGGTGTTGACCCATTGTT** |
| **tyrp-1** | **AAGTTCAATGGCCAGGTCAG** | | **TCAGTGAGGAGAGGCTGGTT** |
| **mitf** | **GGAACAGCAACGAGCTAAGG** | | **GGAACAGCAACGAGCTAAGG** |
| **silv** | GGCACACACACAATGGAAGT | | **AGGAAGTGCTTGGTCTCTCC** |
| **gapdh** | **AACTTTGGCATTGTGGAAGG** | **ACACATTGGGGGTAGGAACA** | |


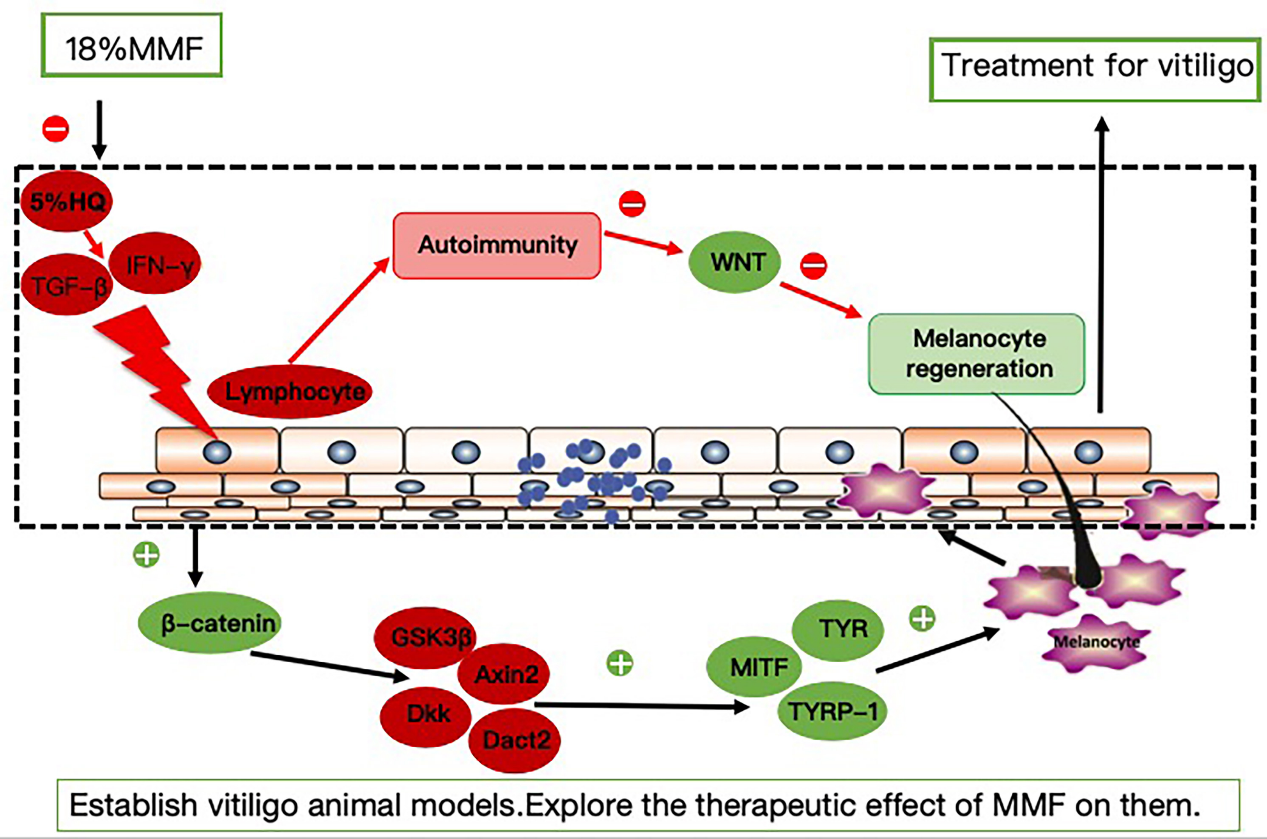


Supplementary Figure 1: (a) The flow chat of the experiment.


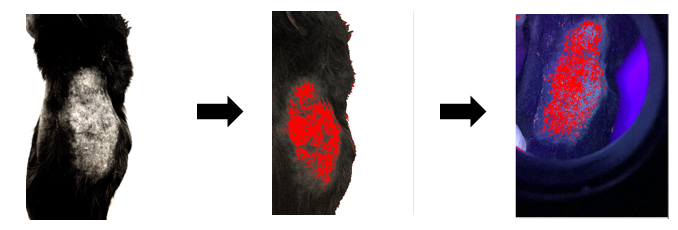


Supplementary Figure 2: (a) Calculation of the percentage of decolourizated area on the hair-removed skin by the Image-Pro Plus.
